# Prevalence and Prognosis of Anemia in Dogs with Degenerative Mitral Valve Disease

**DOI:** 10.1155/2016/4727054

**Published:** 2016-10-20

**Authors:** Ivarosa Bing-Ye Yu, Hui-Pi Huang

**Affiliations:** Institute of Veterinary Clinical Science, Veterinary School, National Taiwan University, No. 1, Section 4, Roosevelt Road, Taipei 100, Taiwan

## Abstract

In humans, heart failure (HF) and renal insufficiency (RI) have negative reciprocal effects, and anemia can exacerbate their progression. In this retrospective study, the prevalence and prognostic significance of anemia in 114 dogs with degenerative mitral valve disease (DMVD) was investigated. Pretreatment clinical parameters, prevalence of anemia and azotemia, and survival time were analyzed in relation to HF severity. The prevalence of anemia was highest in dogs with the modified New York Heart Association (NYHA) class IV HF (33.3%), followed by classes III (15.2%) and II (0%;* p* < 0.001). The presence of anemia was associated with HF severity and blood creatinine > 1.6 mg/dL (both* p *< 0.001). Anemic dogs had a shorter median survival [13 months; 95% confidence interval (CI): 0.7–19.1] than nonanemic dogs (28 months; 95% CI: 15.3–40.7;* p* < .001). NYHA class IV (hazard ratio (HR): 3.1, 95% CI: 2.2–4.3;* p* < 0.001), left atrium/aorta ratio > 1.7 (HR: 2.7, 95% CI: 1.7–4.2;* p* = 0.001), and presence of anemia (HR: 1.43, 95% CI: 1.1–1.9;* p* = 0.004) emerged as predictors of mortality. A cardiorenal-anemia syndrome-like triangle was observed and anemia was a prognostic factor for survival in dogs with DMVD.

## 1. Introduction

There is growing awareness of an association between chronic heart failure (HF) and renal insufficiency (RI) in dogs [1, 2]. The prevalence of azotemia is elevated in dogs with chronic heart valve disease and the risk of azotemia increases with HF severity [1]. In humans, renal dysfunction is a critical independent risk factor of poor outcome and mortality in patients with HF [3, 4].

Comorbid HF, RI, and anemia form a clinical triangle termed cardiorenal-anemia syndrome wherein HF and RI have negative reciprocal effects, and their mutual exacerbation is aggravated by anemia [5]. Anemia has been found to be a common comorbidity in human patients with HF and its presence not only is associated with worse long-term HF outcomes [6, 7], but also is a marker of subclinical comorbid RI [8]. Anemia and RI have an addictive effect on mortality and are independent risk factors for mortality in human patients with HF [9, 10].

Anemia is generally considered to be prevalent in human patients with HF, though prevalence rates in the literature vary widely, ranging from 9.9% to over 50% [11, 12]. Severity of anemia tends to increase in parallel with severity of New York Heart Association (NYHA) functional status [12, 13]. The pathophysiology relating anemia to HF in humans is multifactorial, including renal dysfunction and impaired erythropoietin production [5], overproduction of proinflammatory cytokines such as tumor necrosis factor and interleukins [14, 15], an expansion in plasma volume [16], and downregulation of erythropoietin, such as by angiotensin-converting enzyme inhibitors [17].

Though less well studied in dogs than in humans, a pattern of interactions similar to that seen in human patients appears to be at work. Nicolle and coauthors found that 50% of a group of 124 dogs with chronic heart valve disease had concomitant azotemia and that severity of azotemia and RI increased with severity of HF [1]. Meanwhile Slupe and coauthors found that 28% of a group of 116 dogs with HF presented with anemia characterized by low hematocrit and hemoglobin (Hb) concentrations [18].

The association among HF, impaired renal function, and anemia has not been well studied in dogs. Although treatment for dogs with HF has improved substantially in recent decades, it is not clear how azotemia and anemia affect survival in dogs with HF. The aims of this study were to evaluate the associations of pretreatment hematological [Hb concentration and packed cell volume (PCV)] and biochemical [blood urea nitrogen (BUN) and creatinine concentrations] parameters with survival in dogs with chronic degenerative mitral valve disease (DMVD). We hypothesized that survival time would be shortened in the presence of anemia and azotemia.

## 2. Materials and Methods

### 2.1. Animals

The medical records of 1,188 dogs examined at the Cardiology Unit of the National Taiwan University Veterinary Hospital between 2006 and 2015 were reviewed. DMVD cases were compiled according to the following inclusion and exclusion criteria described below. The inclusion criterion was a first-time diagnosis of DMVD based on clinical presentation and findings of physical, thoracic radiographic, and echocardiographic examinations. The diagnosed criteria of DMVD were based on echocardiographic findings: 2D detection of mitral valve prolapse; any degree of mitral valve leaflet thickening or both; color Doppler identification of any degree of mitral valve regurgitation [19]. The exclusion criteria were DMVD without presenting with clinical signs and no cardiac remodeling based on echocardiographic findings (no enlarged left atrium, left ventricle or both, and left atrium to aorta ratio [LA/Ao] < 1.4) [19, 20]. DMVD presented clinical signs and LA/Ao > 1.4 in echocardiographic examination but did not receive any form of treatment for HF, any other cardiovascular disorders, vector-borne diseases, acute/chronic renal failure, or other systemic disorders (gastrointestinal, hepatic, and neoplastic), having been treated with potentially nephrotoxic drugs.

All dogs in this study receive standard medical treatment for HF, including angiotensin-converting enzyme inhibitor, furosemide, digoxin, and pimobendan based upon the clinical signs and chest radiographic and echocardiographic findings [20].

### 2.2. Study Design

#### 2.2.1. Association of Variables with HF Severity

The study cases were divided into groups based on the NYHA classification system and averaged study variables were compared across the NYHA class groups. The following pretreatment clinical information was collected from the case records of enrolled subjects: signalment; clinical presentation; results of physical examinations, including systemic arterial blood pressure; blood test results, such as Hb concentration, PCV, and blood concentrations of albumin, BUN, and blood creatinine; and urinalysis results, including microscopic examination of urinary sediments and urine specific gravity (refractometric method). Additionally, vertebral heart scale was measured using electronic calipers in the right lateral recumbent view of digital thoracic radiographs [21], and left atrium to aorta ratios (LA/Ao, measured in the 2D right parasternal short-axis view) were compared across the NYHA class groups [19].

In this study, anemia was defined as Hb < 12.5 g/dl and/or PCV < 35%. Azotemia was defined as BUN > 26 mg/dL and/or blood creatinine > 1.6 mg/dL.

#### 2.2.2. Survival

Clinical progression was assessed by review of medical records. Survival time was counted from the day of DMVD diagnosis to either the month of death or closing time of the study (May 31, 2015). When the date of death was not available in the medical record, the dog's owner was contacted to obtain this information. The end-point of the study was death (all causes). All owners of dogs whose cases were included in this study gave their informed consent. The study was carried out in accordance with the code of the National Taiwan University.

### 2.3. Data Analysis

Continuous data with normal distributions are presented as means ± standard deviations, and skewed data are presented as medians (ranges). The homogeneity of variance of each group was checked with the Levene test. Group comparisons of blood and urine test results were made with analyses of variance and Scheffé's* post hoc *pairwise comparisons. Categorical variables (i.e., absence or presence of anemia, azotemia) were evaluated with Pearson's chi square test.

Kaplan-Meier survival curves were created to identify potential prognostic variables for death by any cause (all-cause mortality) and log-rank tests were used to assess significance. Dogs that were alive at the time of data analysis were censored in the survival analysis, and the last known date alive was used for dogs lost to follow-up. A univariate analysis was conducted to assess predictors (e.g., NYHA class IV, LA/Ao > 1.7, presence of anemia, presence of azotemia, and blood creatinine > 1.6 mg/dL) of all-cause mortality. Risks were quantitated as hazard ratios (HRs) with 95% confidence intervals (CIs). Statistical analyses were performed with a commercial statistical software package. Two-tailed* p* values < 0.05 were considered significant.

## 3. Results

### 3.1. Cohort Characteristics

A total of 114 dogs (61 females) met the inclusion criteria and were included in the study. Their mean age at the time of DMVD diagnosis was 11.1 ± 3.2 years. Sixteen breeds were represented in the cohort, with the most represented breed being Maltese Terrier. Thirty-eight dogs were categorized as the NYHA class II, and 46 and 30 dogs were categorized as the NYHA classes III and IV, respectively (Table 1).

### 3.2. Prevalence of Anemia and Azotemia

Hb concentration, PCV, and RBC were highest in the NYHA class II (16.9 ± 2.0 g/dL, 47.1 ± 5.5 %, and 7.2 ± 1.0 × 10^6^ /*μ*L, resp.), followed by classes III (14.5 ± 2.8 g/dL, 39.6 ± 8.1 %, and 6.1 ± 1.2 × 10^6^/*μ*L, resp.) and IV (13.8 ± 2.9 g/dL, 37.8 ± 7.9 % and 5.9 ± 1.4 × 10^6^/*μ*L, resp.;* p* < 0.001,* p* < 0.001, and *p* < 0.001, resp., Table 2). The overall prevalence of anemia in dogs with DMVD was 14.9%, with prevalence showing an increasing trend from class II (0%) to class III (15.2%) to class IV (33.3%,* p* < 0.001, Table 2). The presence of anemia was positively associated with severity of NYHA functional classification and blood creatinine > 1.6 mg/dL (both* p *< 0.001), but not with the presence of azotemia (*p* = 0.074).

BUN and blood creatinine concentrations were highest in class IV (50.8 ± 17.8 and 1.4 ± 0.9 mg/dL), followed by classes III (37.3 ± 14.9 and 1.1 ± 0.6 mg/dL) and II (26.5 ± 15.8 and 0.9 ± 0.4 mg/dL;* p* = 0.032 and* p* = 0.018, resp., Table 2). The overall prevalence of azotemia in dogs with DMVD was 44.7%, highest in the NYHA class IV (63.3%), followed by classes III (41.3%) and II (34.2%), but the differences among three classes were not significant (*p* = 0.104, Table 2). The overall prevalence of blood creatinine > 1.6 mg/dl was 14.9%, highest in the NYHA class IV (23.3%), followed by classes III (17.4%) and II (5.3%). The prevalence of blood creatinine > 1.6 mg/dL among three groups was significantly different (*p* = 0.035, Table 2).

### 3.3. Survival Analysis

**Figure 1:**
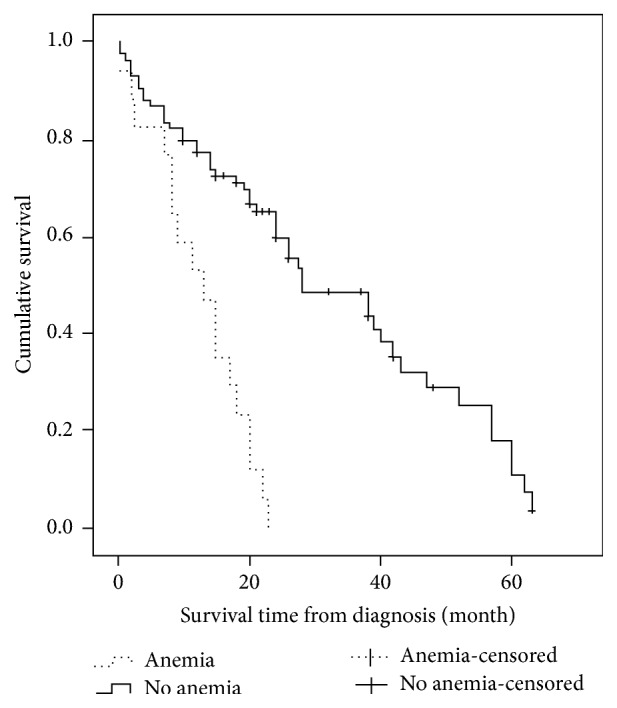
Kaplan-Meier curves demonstrating survival of 101 dogs newly diagnosed with degenerative mitral valve disease and categorized by pretreatment anemia status (hemoglobin concentration < 12.5 g/dl and/or packed cell volume < 35%). Data for dogs that were still living (*n* = 34) at the conclusion of the study were censored (hatch marks).

Of the 114 DMVD dogs, 101 were able to complete the follow-up and 34 were still alive at the conclusion of the study, and data regarding these dogs were censored from the survival analysis. The median survival time was 24 months (95% CI: 18.5–29.5). The median survival times for classes II, III, and IV were 42 months (95% CI: 33.7–50.3), 26 months (95% CI: 21.3–30.7), and 7 months (95% CI: 3.6–10.4), respectively (*p* < 0.001, Table 1). The median survival time for dogs without anemia was 28 months (95% CI: 15.3–40.7) and for those with anemia was 13 months (95% CI: 0.7–19.1;* p* < 0.001; Figure 1). The median survival time for dogs without azotemia was 39 months (95% CI: 20.4–57.5) and for those with azotemia was 20 months (95% CI: 14.5–25.5;* p* = 0.173). The median survival time for dogs with blood creatinine ≤ 1.6 mg/dL was 43 months (95% CI: 18.2–67.5) and for those with blood creatinine > 1.6 mg/dL was 20 months (95% CI: 10.5–29.5;* p* = 0.159).

A univariate analysis indicated that designation to the NYHA class IV group (HR: 3.1, 95% CI: 2.2–4.3;* p* < 0.001), LA/Ao > 1.7 (HR: 2.7, 95% CI: 1.7–4.2;* p* = 0.001), and presence of anemia (HR: 1.43, 95% CI: 1.1–1.9;* p* = 0.004) were significant predictors of all-cause mortality. Meanwhile, presence of azotemia and blood creatinine > 1.6 mg/dL were not predictors of all-cause mortality in the univariate analysis.

## 4. Discussion

In this study, we found that Hb concentration and PCV decreased with increasing severity of NYHA functional status in dogs with DMVD. Meanwhile, BUN levels, blood creatinine levels, prevalence of anemia, and prevalence of blood creatinine > 1.6 mg/dL increased with increasing HF severity. Anemia was associated with a poor outcome and emerged as a predictor of mortality.

The present findings fit with findings in human patients indicating that the prevalence of anemia comorbidity with HF increases with increasing severity of NYHA functional status and that anemia is a prognostic factor for mortality [12, 13]. The overall prevalence of anemia of this study (14.5%) was lower than that reported previously (28%) [18]; however, an increasing prevalence of anemia was in relation to greater HF severity, with the rate reaching 33.3% in the NYHA class IV group.

Although the precise pathophysiology of anemia in dogs with DMVD is not yet known, it is worth noting that dogs with disorders that could potentially cause anemia independent of HF, such as tick-borne diseases, chronic renal failure, and neoplasia [22], were excluded from this study. Likewise, dogs with systemic diseases that may cause iron deficiency, such as chronic enteropathy, were also excluded.

Our findings of higher BUN levels, blood creatinine levels, and prevalence of blood creatinine > 1.6 mg/dL among dogs with advanced HF are consistent with previous studies [1, 23]. None of the dogs in this study suffered from acute or chronic renal disorders, and none of them presented with low urine specific gravity. Thus, the presence of azotemia was presumably secondary to HF [1] even though the comparison of azotemia prevalence across the NYHA class groups did not reach significance.

To the best of our knowledge, this is the first study to demonstrate an effect of anemia on survival in dogs with HF in the veterinary literature [18]. We found that prognosis was less favorable for anemic than for nonanemic dogs and that a pretreatment Hb concentration < 12.5 g/dL was a predictor of poor outcome. These results are consistent with studies of human HF patients in indicating that anemia is an independent predictor of mortality in HF patients [7, 13, 24]. Correction of anemia is strongly recommended to improve outcomes in human patients with HF [24]. Studies of the effectiveness of anemia correction for improving outcomes in dogs with DMVD are needed.

The present findings showing that severity of HF at admission and LA/Ao > 1.7 were also predictors of poor outcome in dogs with DMVD are consistent with the results of previous studies [25, 26]. Although the blood test result of blood creatinine > 1.6 mg/dL was more prevalent in severe HF cases, relative to less severe cases, it was not significantly associated with a poor outcome nor was it a predictor of mortality.

In this study, the presence of anemia was positively associated with both HF severity and blood creatinine > 1.6 mg/dL, a sign of development of RI. Blood creatinine > 1.6 mg/dl was also more prevalent in the dogs with advanced HF. The associations among HF, RI, and anemia observed in this cohort are consistent with so-called “cardiorenal-anemia syndrome” described in humans [5] and support the notion that there are cause and effect relationships among HF, RI, and anemia in dogs with DMVD.

This study has some limitations worth noting. We did not have control comparison for the prevalence of anemia and azotemia, and we did not have an age-matched healthy control group with which to compare survival times.

## 5. Conclusions

Pretreatment anemia, as indicated by Hb < 12.5 g/dL, at the time of diagnosis of DMVD is predictor of poor outcome in dogs. Anemia also emerged as a prognostic factor for survival in dogs with DMVD. A cardiorenal-anemia syndrome-like interrelationship of clinical parameters can be seen in dogs with DMVD.

## Figures and Tables

**Table 1:** Baseline characteristics of 114 dogs affected with degenerative mitral valvular disease according to the modified New York Heart Association (NYHA) functional classification.

Characteristic	NYHA II (*n* = 38)	NYHA III (*n* = 46)	NYHA IV (*n* = 30)
Age, years	9.5 ± 3.5	11.6 ± 2.7^†^	12.3 ± 3.1^†^
Number of females/number of males	24/14	22/24	15/15
Body weight, kg	5.4 ± 2.4	5.8 ± 3.7	5.3 ± 2.7
Heart rate, beats per minute	114 ± 26	131 ± 26^†^	135 ± 27^†‡^
Blood pressure, mmHg	142 ± 23	136 ± 23	122 ± 19^†^
Vertebral heart scale	9.9 ± 0.9	10.9 ± 0.5^†^	12.5 ± 0.4^†‡^
LA/Ao	1.6 ± 0.3	1.9 ± 0.3^†^	2.5 ± 0.4^†‡^
Median survival (95% CI), months	42 (33.7–50.3)	26 (21.3–30.7)	7 (3.6–10.4)

Data are presented as mean ± SD or median (95% confidence interval [CI]).

LA/Ao, left atrium to aorta ratio.

^†^ *p* < 0.05 versus class II; ^‡^ *p* < 0.05 versus class III.

**Table 2:** Clinical test results in 114 dogs with degenerative mitral valvular disease according to the modified New York Heart Association (NYHA) functional classification.

Parameter	NYHA II (*n* = 38)	NYHA III (*n* = 46)	NYHA IV (*n* = 30)
Hb, g/dL	16.9 ± 2.0	14.5 ± 2.8^†^	13.8 ± 2.9^†^
Packed cell volume, %	47.1 ± 5.5	39.6 ± 8.1^†^	37.8 ± 7.9^†^
RBC count, 10^6^/*µ*L	7.2 ± 1.0	6.1 ± 1.2^†^	5.9 ± 1.4^†^
Prevalence of anemia	0% (0/38)	15.2% (7/46)	33.3%^†^ (10/30)
Albumin, g/dL	3.3 ± 0.5	3.1 ± 0.5	3.1 ± 0.4^†^
BUN, mg/dL	26.5 ± 15.8	37.3 ± 14.9	50.8 ± 17.8^†^
CRE, mg/dL	0.9 ± 0.4	1.1 ± 0.6	1.4 ± 1.0^†^
Prevalence of azotemia	34.2% (13/38)	41.3% (19/46)	63.3% (19/30)
Prevalence of CRE > 1.6 mg/dL	5.3% (2/38)	17.4% (8/46)	23.3%^†^ (7/30)
Urine specific gravity	1.028 ± 0.011	1.025 ± 0.012	1.023 ± 0.009

Data are presented as mean ± SD or % (*n*).

BUN, blood urea nitrogen; CRE, blood creatinine; Hb, hemoglobin; RBC, red blood cell.

^†^ *p* < 0.05 versus class II.
